# Coalescence and Collisions of Gold Nanoparticles

**DOI:** 10.3390/ma4020368

**Published:** 2011-01-28

**Authors:** Joel Antúnez-García, Sergio Mejía-Rosales, Eduardo Pérez-Tijerina, Juan Martín Montejano-Carrizales, Miguel José-Yacamán

**Affiliations:** 1 CICFIM, Facultad de Ciencias Físico-Matemáticas, Universidad Autónoma de Nuevo León, San Nicolás de los Garza, N.L. 66450, Mexico; 2 Department of Physics and Astronomy, University of Texas at San Antonio, San Antonio, TX 78249, USA; 3 Centro de Innovación, Investigación y Desarrollo en Ingeniería y Tecnología (CIIDIT), Universidad Autónoma de Nuevo León, Apodaca, Nuevo León 66600, Mexico; 4 Instituto de Física, Universidad Autónoma de San Luis Potosí, San Luis Potosí, S.L.P. 48000, Mexico; 5 Centro de Nanociencias y Nanotecnología, Universidad Nacional Autónoma de México, Ensenada, B.C. 22800, Mexico

**Keywords:** gold nanoparticles, coalescence, collisions, molecular dynamics, common neighbor analysis

## Abstract

We study the assembling of small gold clusters subject to collisions and close contact coalescence by using molecular dynamics simulations to simulate events that occur typically in the sputtering process of synthesis. Our results support the notion that the kinetics of coalescence processes strongly determine the geometry and structure of the final particle. While impact velocities, relative orientations, and the initial shape of the interacting particles are unlikely to strictly determine the structural details of the newly formed particle, we found that high initial temperatures and/or impact velocities increase the probability of appearance of icosahedral-like structures, Wulff polyhedra are likely to be formed as a product of the interactions between nanospheres, while the appearance of fcc particles of approximately cuboctahedral shape is mainly due to the interaction between icosahedra.

## 1. Introduction

It is known that the physical and chemical properties of metallic clusters can be substantially different from those of the same material at the bulk [1]. Thus, the optical response of silver and gold nanoclusters and small nanoparticles depend strongly both on the size and the shape of the structure [2,3], and the chemical affinity of metal nanoparticles. Their catalytic activity for specific reactions is also determined by the dimensions and the details of the surface of the particles [4]. The understanding of the processes of formation of metal nanoparticles may contribute to finding optimal approaches to control the size and shape of the particles, and to the fine-tuning of these properties to produce particles for specific functional applications.

Currently, it is possible to produce metal nanoparticles by a broad diversity of experimental techniques, obtaining very narrow size distributions and specific mean sizes by the control of a few parameters. In particular, physical methods of production of nanoparticles such as inert gas condensation allow a very precise control on the number of atoms constituting the particles, by the appropriate choice of partial gas pressure, voltage, and length of the active region of synthesis [5]. Additionally, the use of physical methods assures the production of nanoparticles with well-defined geometries. Nevertheless, the physical mechanisms involved in the synthesis of metal particles are far to be completely understood. Baletto *et al.* [6] have studied the process of attachment of individual atoms to a small cluster acting as a seed for the growth of a nanoparticle, and Grochola *et al.* have studied the formation of gold clusters on vapor synthesis at several initial growth conditions [7]. In an inert gas condensation process, the attachment of atoms, coagulation, and coalescence, occur at different rates, depending on the synthesis conditions, and, if the temperature is not very low, coalescence processes will predominate [5]. Furthermore, once the particles are formed in the synthesis chamber and deposited in a substrate, coalescence may occur between two particles close to each other, or between a particle that lands into the substrate close to another particle already deposited. Mariscal *et al.* [8] have studied these processes using Molecular Dynamics simulations in the particular case of collisions between metal particles of different compositions, finding that both the chemical elements and the velocity of collision have a determining effect on the final structure of the newly formed particle. A second study by Mariscal *et al.* [9] confirms the role of initial velocity as a control parameter in collisions, and reinforces the generally accepted idea that kinematic processes have a determining role in the final structure of the particles.

Despite of the different experimental arrangements used to produce metal nanoclusters and nanoparticles, several structures are obtained which are common to all the techniques. For fcc metals, the most common structural motifs described in literature are icosahedral ($I_{h}$), and decahedral ($D_{h}$) [10,11,12,13], and multiple twinned particles, either icosahedral or decahedral, are commonly observed [14]. Different theoretical studies show that the decahedral structures are energetically more favorable than the icosahedra, but different experimental results show that gold nanoparticles grow preferentially as icosahedra, at sizes as large as 43 nm [15], and even icosahedral particles larger than 50 nm have been produced [16,17]. From these results it can be inferred that the finite temperature kinetic effects strongly determine the final shape of the particles [12,18,19,20,21,22]. Long nanorod-like shapes can also be produced with relative simplicity, although at the present the formation mechanism is not clearly understood [23].

This work takes into consideration the third mechanism involved in the synthesis of metal nanoparticles by physical methods, namely, the coalescence of particles of similar sizes. To address this issue, we performed a set of Molecular Dynamics simulations of the interaction between two gold nanoparticles when the relative velocity is zero (we will refer to these processes simply as *coalescence*), and when the particles come into contact at a finite relative velocity (which we will call indistinctly *impacts* or *collisions*). The paper is organized as follows. Section 2 describes the starting structures, the parameters and interaction model used in the simulations, and the conditions at which the simulations are performed. In Section 3 the results of the simulations are analyzed and discussed. Finally, we summarize some of the main points of this investigation in Section 4, and make some remarks in relation to future work.

## 2. Description of the Simulations

**Figure 1:**
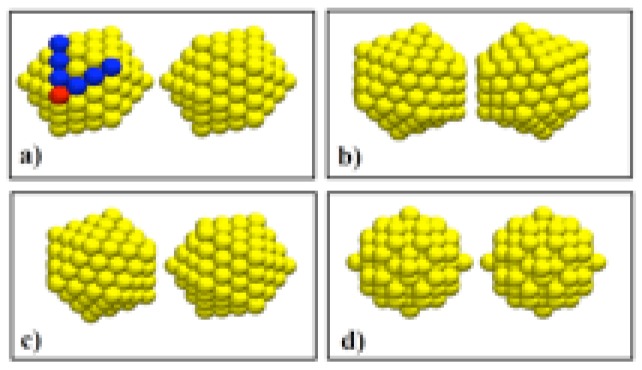
Different initial configurations used in the MD simulations. (**a**) $I_{h}$, vertex-to-vertex; (**b**) $I_{h}$, facet-to-facet; (**c**) $I_{h}$, facet-to-vertex; (**d**) fcc quasi-spheres. The darker atoms on (a) define the order *ν* of the icosahedron; for an $I_{h}$ of 147 atoms as the one shown in the figure, ν=3.

The particles used to simulate the coalescence and impact processes were of two different geometries: icosahedra of 147, 309, 561, and 923 atoms, and spherical clusters (extracted from a fcc lattice) of 141, 300, 500 and 900 atoms. The slight difference in the number of atoms of the spheres with respect of those of the icosahedra of similar size was due to the restriction imposed by the geometry and the symmetry of the spherical clusters. Four different configurations, each consisting in a pair of particles, were considered for the coalescence simulations: Two icosahedra at three different relative orientations, and two spherical particles. These configurations are shown Figure 1 for the smallest particles. The configurations were thermalized independently at the appropriate temperature, using constant temperature molecular dynamics runs for a time large enough to stabilize the average configurational energy. Along the thermalization process, the particles were kept separated a distance large enough to avoid interactions between atoms of different particles. Once the particles were thermalized, nine typical configurations were chosen for each system to be the starting points of the coalescence simulations. The simulations were made at three different initial temperatures $T_{i}$: 300, 350 and 400 K. For the simulations of collisions between clusters, the velocity distribution on each cluster was modified by adding an impact velocity along one direction. Four different relative velocities $V_{r}$ were used: 0 (with the particles almost at contact), 200, 300, and 500 m/s. The range of velocities was chosen such that the conversion of kinetic into thermal energy will not melt the resulting structure, following previous results from our group of MD simulations of heating of gold clusters, [24] where the melting transitions were located by analyzing the behavior of the global order parameter $Q_{6}$, as defined by Chushak and Bartell. [25] Considering the different geometries, initial configurations, temperatures, and velocities, a total of 432 production runs were performed for each size. We concentrated our analysis on those runs where the resulting final particles have recognizable shapes and structures.

The XMD [26] code was employed to carry out our simulations. The interactions between atoms were modeled using the embedded atom (EAM) potential [27]. The equations of motion were integrated using a fifth-order gear predictor-corrector algorithm at every time step of 2 fs. After equilibration, the trajectory for every production run was 5 ns long.

## 3. Results

**Figure 2:**
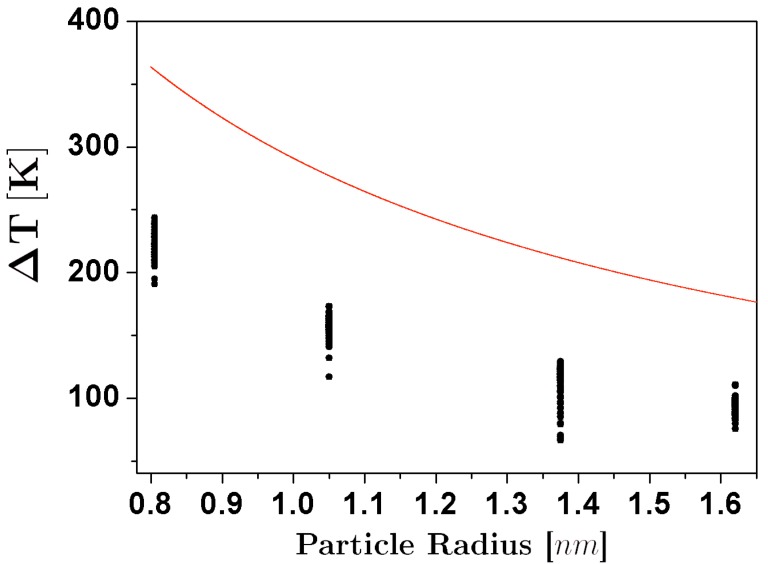
Temperature increase due to the coalescence of icosahedral nanoparticles, as a function of the size of the coalescing particles. The dots represent the simulations results, and the dotted line is the expected behavior of Δ(T), assuming sphericity and bulk values of surface tension, heat capacity, and density.

The coalescence of two particles into a new larger particle lowers the surface energy, and hence an increment in temperature is expected. The temperature rise has been described by Equation 1, that takes into consideration the surface energy differences, and assumes that the initial and final particles are approximately spherical [28]:

(1) $$\Delta \left(T\right)=C\frac{1}{R_{2}}\frac{\left[1+{\left(R_{1}/R_{2}\right)}^{2}\right]-{\left[1+{\left(R_{1}/R_{2}\right)}^{3}\right]}^{2/3}}{\left[1+{\left(R_{1}/R_{2}\right)}^{3}\right]}$$

here, $R_{1}$ and $R_{2}$ are the radii of the original particles, and *C* is a constant that depends on the surface tension, the heat capacity, and the density of the material, that was adjusted using reported bulk values of these quantities. The results for Δ(T) in processes of coalescence of icosahedra, and their comparison with the estimate of Equation 1 are shown in Figure 2. From the comparison it is evident that even if there is a considerable increase of temperature at all the particle sizes, the temperature rise falls short of the theoretical estimates by more than 100K. This can be explained by the fact that neither the initial structures nor the final particle are strictly spherical, and thus the conversion of surface energy into thermal energy is less than the expected for the case of nanospheres. On the other hand, we can note that the differences between the simulation results and the theoretical estimate become less pronounced as the size of the particle increases, a behavior that is not unexpected, considering that the adjust of the *C* parameter was made from bulk values. Even for the smallest sizes considered in this study, the increase in temperature was not high enough to melt the structure of the particles.

**Figure 3:**
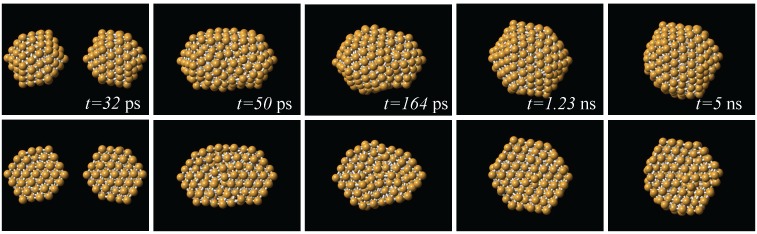
Series of snapshots of the collision between two 147-atom icosahedra. The two particles impact to each other with two of their vertices aligned to the impact direction, and the resulting structure is an $I_{h}$ an order larger than the original ones. Upper row: complete structures; lower row: cross sections.

From the production runs we observed that in both coalescence and collision processes, one of the effects of increasing the initial temperature is to increase the probability of appearance of icosahedral-like structures. This is also the case when the relative velocity of collision is increased. Globally, the dominant structural motif was of the icosahedral kind. This is exemplified in Figure 3, where is shown a series of snapshots of the interaction between two 147-atom icosahedra. Here, the impact velocity was 200 m/s, and the initial temperature was 300 K; after the impact, the temperature of the resulting particle was 568 K. The two particles impact to each other with two of their vertices aligned to the impact direction. The final particle produced by this process is also icosahedral, an order larger than the original impacting particles. From Figure 3 we can also see that the reorganization of the atoms to form the new particle occurs on the first nanosecond of the simulation. In almost all of the simulation runs, the dynamics that allowed to reach an stable final structure, except for possible regions in the structure that kept a liquid-like state along the whole run, occurred during the first of the 5 nanoseconds of dynamics.

**Figure 4:**
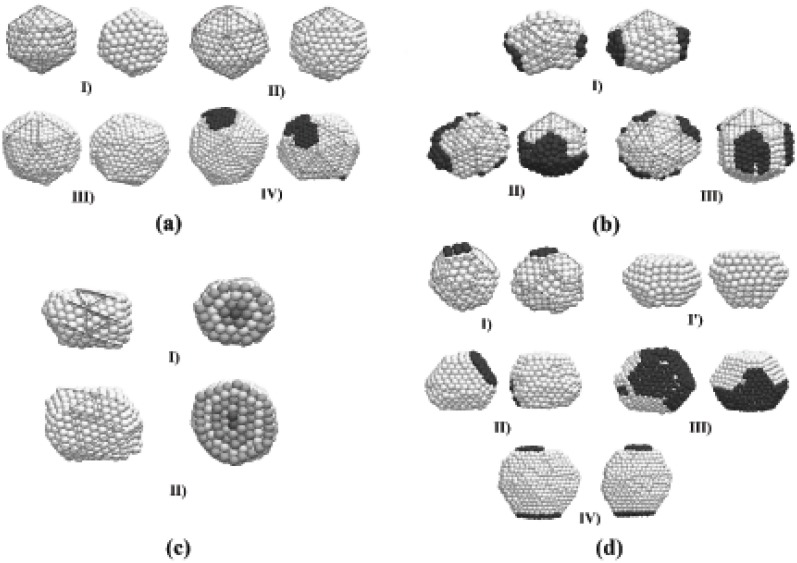
Final structures obtained through the self-assembling of two gold clusters, shown at two different orientations. (**a**) Icosahedral kind (Ih) structures of order 4, 5, 6, and 7; (**b**) Stellated Marks decahedra (m-Dh), surrounded by atoms (dark color) along the (100) planes; (**c**) Helical particles made of coaxial shells (different colors have been used to emphasize the shells structure); (**d**) Truncated octahedral-like (TO) structures.

Despite the fact that the preferential final structure was $I_{h}$, other structural motifs were obtained. Wulff polyhedra are likely to be formed as a product of the interactions between spheres, while fcc particles of indefinite shape are mainly due to the interaction between icosahedra. A possible explanation to this is that the $I_{h}$ geometry is energetically more stable than a spherical shape, and thus the potential energy barrier that must be surpassed to obtain a truncated octahedron TO structure is higher when the initial particles are have an $I_{h}$ structure. The details of the final shape and structure of the particles may vary, even for the simulations performed under the same conditions, although some main features are shared between particles of different shapes. We show in Figure 4 several representative final structures, for purposes of discussion of their structural features. Each configuration is shown at two orientations. The particles of Figures 4(a) have icosahedral ($I_{h}$) structures, where well-defined (111) facets are observed. The particles of Figures 4(b) are structures that can be thought of as decorated Marks decahedral shapes (*m*-$D_{h}$, according to the notation employed by Cleveland *et al.* [22]); the *m*-$D_{h}$ (342) decahedron becomes clear when some atoms at the surface (distinguished by color in the figure) are removed from the particle. In order to make a distinction between the obtained structures from the ideal Ih and *m*-$D_{h}$ (342), we will refer to the structures of the kind shown in Figure 4 as *q*-$I_{h}$ and *q*-$D_{h}$, respectively. Figure 4(c) show particles of truncated octahedron-like (TO) shape. The particles of Figure 4(d) have helical-like structure, formed by concentric shells of stripes. These helical particles have a comparatively low probability of appearance but, once they are formed, these structures are stable enough to survive along the whole simulation. The dynamics and structural detail of these particles are quite peculiar and a detailed analysis will be discussed in an oncoming article.

The number of atoms that complete a perfect icosahedron (called *magic number*) is given by [29]

(2) $$M_{N}\left(\nu \right)=\left(10/3\right)\nu^{3}+5\nu^{2}+\left(11/3\right)\nu +1$$

where *ν*, the order of the icosahedron, is given by the number of icosahedral shells surrounding a central atom, or, as can be noted in Figure 1(a), by the number of border atoms shared by two (111) faces in the surface of the icosahedron. In the case of the coalescing icosahedra of Figure 1, ν=3. In the runs where *q*-$I_{h}$ structures were generated, the structures assemble themselves into the *q*-$I_{h}$ geometry of the magic number closest to their number of atoms. This can be noted for example in the last snapshot of Figure 3, where the total number of atoms is 618, and the order of the *q*-$I_{h}$ is ν=5, which corresponds to a $M_{N}\left(5\right)$ of 561. On the other hand, the smallest *q*-$I_{h}$ structures obtained with the simulations were of order ν=4, although the total number of atoms in the configurations (282 for two coalescing icosahedra, 294 for two spheres) are less than $M_{N}\left(4\right)=309$.

We can also note in Figure 4 (b) that when the resulting particle has a *q*-$D_{h}$ geometry, the (111) surface planes are an order less than those formed in the surface of the *q*-$I_{h}$ particles of the same size. The *q*-$D_{h}$ particles are decorated by the remaining atoms with a distribution that makes the particle to take a star-like shape. Different stellations of $D_{h}$ metallic particles have been observed experimentally and described in detail, but at sizes considerably larger than those used in this work, and the formation mechanisms may not be the same of the smaller particles. The 6 surface stripes of the first helical particle of Figure 4(d) can be constructed by rolling triangular planes of the same order than the *m*-$D_{h}$ structure. In a small number of cases, during the self-assembling we observed a particle sharing icosahedral and decahedral structural domains. Generally this situation conducted eventually to a final *q*-$I_{h}$ structure, but sometimes this structural arrangement survived along the whole time of the simulation. Experimental evidence of this structural coexistence has been recently provided by Koga [30].

**Figure 5:**
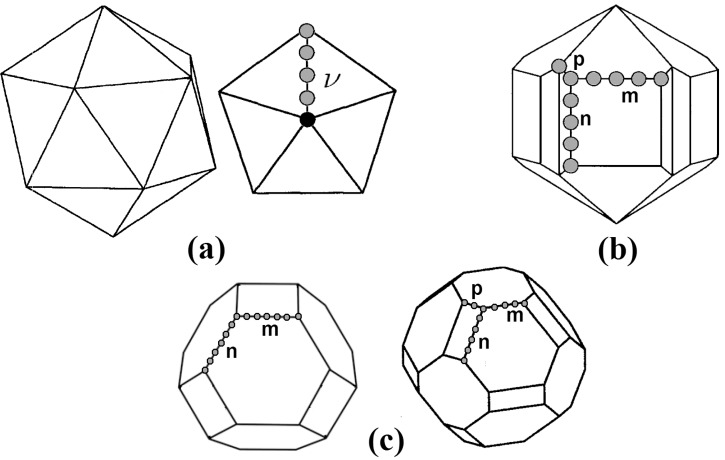
(**a**) ideal $I_{h}$ geometry. The atoms shared between two triangular facets define the order *ν* of the icosahedron; (**b**) n×m×p Marks decahedron, characterized by the v-shaped cuts of depth *p* into the edges of the n×m facets; (**c**) Two kinds of n×m×p truncated octahedra.

The main geometric features of the most probable final configurations are described in Figure 5. In *(a)*, an ideal $I_{h}$ geometry is sketched, where the spheres representing the atoms shared between two triangular facets define the order *ν* of the icosahedron. For the *q*-$I_{h}$ structures shown in Figure 4 *(a)*, the values of *ν* are 4, 5, 6, and 7, respectively. The sketch of Figure 5 *(b)* represents a n×m×p Marks decahedron, characterized by the v-shaped cuts of depth *p* into the edges of the n×m facets. The *q*-$D_{h}$ structures shown on Figure4 *(b)* are stellations of Marks decahedra. Figure 5 *(c)* shows two kinds of n×m×p truncated octahedra, similar to those of the Figure 4 *(c)*.

**Figure 6:**
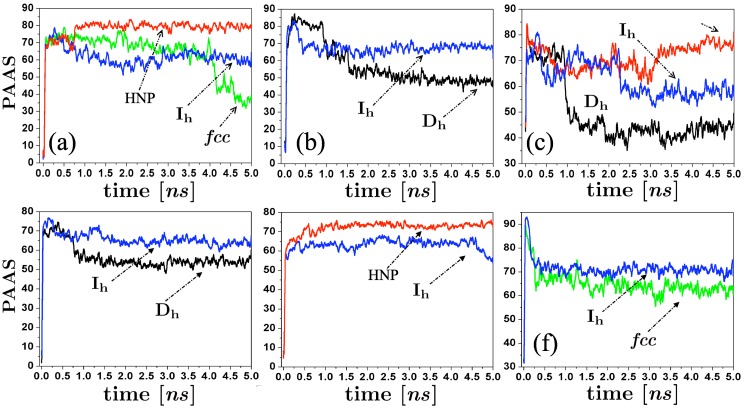
Time evolution of the percentage of atoms in amorphous state along the runs, for some of the structures produced by the simulations ($I_{h}$, $D_{h}$, *fcc*, and helical nanoparticles (HNP)). (**a**) Collisions between 147-atoms icosahedra, vertex-vertex configuration, $T_{i}=300$ K and $V_{r}=200$ m/s; (**b**) Collisions between 147-atoms icosahedra, facet-facet configuration, $T_{i}=350$, $V_{r}=200$ m/s; (**c**) Coalescence between 141-atoms spheres, $T_{i}=300$ K; (**d**) Collisions between 309-atoms icosahedra, vertex-vertex configuration, $T_{i}=300$ K and $V_{r}=300$ m/s; (**e**) Collisions between 309-atoms icosahedra, facet-facet configuration, $T_{i}=350$, $V_{r}=500$ m/s; (**f**) Collisions between 298-atoms spheres, $T_{i}=350$ K, $V_{r}=500$ m/s.

As Hendy and Hall [31] suggest, we employed the common neighbor analysis (CNA) [32], and the Cleveland *et al.* trace analysis [22] to structurally discriminate atoms on amorphous regions of the particle. An atom was considered in an amorphous environment when their atomic coordination is not described at least by a 200 trace. The identity of the atoms on amorphous state and their corresponding configurational energy were monitored along the evolution of the self-assembling process, and the percentage of the energy associated to these atoms was calculated at each time *t*. We plot in Figure 6 the behavior of the percentage of the atoms in amorphous state (PAAS) along the runs, for different initial conditions of our simulations. We can note that even with the atomic reorganization of the spherical clusters after their thermalization process, their associated PAAS values (Figures 6 (c) and (f)) at the very beginning of the simulations is higher than those of the $I_{h}$ clusters. However, this quantity decreases as the size of the spherical cluster increases. We can also note that the appearance of $D_{h}$ and *fcc* structural motifs is characterized by a high decrease of PAAS in comparison with the $I_{h}$ and HNR structural motifs. It should be remarked that, as can be noted at each particular case shown in Figure 6, initial configurations identically prepared (except for different thermalization times) are able to produce different final structural motifs. The energy curves show that at the end of the runs, the m-Dh and FCC clusters reach the lowest configurational energy, since the majority of atoms conforming the structure are on crystallized state.

There are several facts that can be learned through the inspection of the molecular dynamics trajectories. In all the runs where the initial configuration was one of those shown in Figure 1(a-c), when the resulting structure was a *q*-$D_{h}$ or a *q*-$I_{h}$ structure, the new particle was not formed directly by the growth of one of the original icosahedra. Instead, all the atoms get reorganized to build the new particle. Immediately after the original particles got in contact, several (111) faces appear at the surface of the new particle, and, if the initial temperature or the impact velocity is sufficiently high, the extra kinetic energy is enough for the atoms directly below the surface to reorganize themselves to produce a $D_{h}$ particle. In these cases the excess kinetic energy does not produce atomic migrations, the configurational energy is kept low, and the net effect is a high rise on the overall temperature of the decahedra. In addition to the low mobilization of atoms in one direction when a HNP is obtained, we observed that the fast ordering of atoms on the surface allows the formation of helical stripes, followed by an internal reorganization of the particle. This is described by Diao *et al.* [33], in terms of internal compressive intrinsic stress induced by surface stress. Eventually, the surface stress is turned onto tensile stress, and the helical nanostructure is formed. Elongated helical nanostructures have been predicted for Cu [34], Al and Pb[35], and Au [36]. Wang *et al.* [37] predicted that helical nanorods are expected below 3 nm diameter. As in the case of the appearance of FCC structures, the internal organization after the reordering of the surface plays a crucial role on the final shape of the helical particle. A more detailed analysis of these helical structures is in progress and will be the subject of an oncoming article.

## 4. Conclusions

We have used Molecular Dynamics simulations to study the nanostructures resulting from coalescence and impact processes between gold nanoparticles. We have found that, even if the appearance of icosahedral and decahedral particles is likely to occur, neither the relative velocity of impact between particles nor the relative orientation between the impacting particles have a strictly determining effect in the formation of structures with specific shapes. Nevertheless, when the impact velocity or the initial temperature are high enough, the kinetic energy supply given by the restructuring allows the restructuring of the surface of the particles, followed by the reordering of the atoms at the core. For the kind of interactions described here, where the two particles involved in the impacts are of the same size, we found that the construction of $I_{h}$ structures does not happen by the growth of one of the original icosahedra, but the final $I_{h}$ particle is built from a complete reorganization of the atoms of the impacted particles. In addition the $I_{h}$ and $D_{h}$ structures, which are expected to appear, structures formed by concentric shells of helical stripes were also produced by direct impacts between particles. These helical particles are energetically stable enough to survive along the whole trajectory of the simulations. Further work will be centered on making a deep study of these structures, and their possible role as seeds for the growing of helical nanorods.
